# Efficacy and Safety of Surgical Treatment Among Older Adult Patients (80 Years) With Pancreatic Cancer: A Systematic Review and Meta‐Analysis

**DOI:** 10.1002/jhbp.12151

**Published:** 2025-05-14

**Authors:** Naoki Ikenaga, Takao Ohtsuka, Eiji Mitate, Koji Tamura, Masayuki Sho, Masafumi Nakamura

**Affiliations:** ^1^ Department of Surgery and Oncology Graduate School of Medical Sciences, Kyushu University Fukuoka Japan; ^2^ Department of Digestive Surgery Kagoshima University Kagoshima Japan; ^3^ Department of Oral and Maxillofacial Surgery Kanazawa Medical University Ishikawa Japan; ^4^ Department of Surgery Nara Medical University Nara Japan

**Keywords:** non‐surgical treatment, older adults, overall survival, pancreatectomy, pancreatic cancer

## Abstract

**Background:**

With rapid population aging, the number of older adult patients presenting with pancreatic cancer is increasing. This meta‐analysis aimed to clarify the efficacy and safety of surgical interventions for pancreatic cancer among older adult patients.

**Methods:**

Literature published in PubMed, Cochrane Library, and ICHUSHI databases until January 2024 was systematically searched. Comparative studies reporting outcomes of pancreatic cancer resection among patients aged 80 years or older were included in the analysis. Fifteen retrospective studies involving 22 647 patients were included in the meta‐analysis: 2930 patients aged 80 years or older who underwent pancreatic resection, 2059 patients aged 80 years or older treated with chemotherapy, and 17 658 patients under 80 years old.

**Results:**

Surgical treatment had a higher two‐year survival rate than chemotherapy among patients with pancreatic cancer aged 80 years or older. These patients experienced similar morbidity, higher mortality, and lower three‐year survival rates than those younger than 80 years.

**Conclusions:**

Surgical treatment for pancreatic cancer improves survival rates even among patients aged 80 years or older; however, these patients may have a lower chance of recovering from complications. Patients should be informed of these objective findings to ensure shared decision‐making regarding the best treatment strategy.

## Graphical abstract

This meta‐analysis by Ikenaga and colleagues is the first to evaluate surgical treatment for pancreatic cancer in patients aged 80 years or older. Surgical resection improved survival over chemotherapy, despite higher mortality and lower 3‐year survival than in younger patients. Findings highlight surgical benefit and the importance of preoperative evaluation and shared decision‐making.

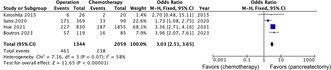

## 1 Introduction

The world is experiencing a rapidly aging population. According to a United Nations report, the percentage of individuals aged 80 years or older will increase from 2% to approximately 5% between 2021 and 2050 [1]. This demographic shift has significant implications for healthcare systems by increasing the incidence of age‐related diseases such as cancer. Pancreatic cancer is one of the most lethal malignancies, with a 5‐year survival rate of approximately 13% [2]. With advances in diagnostic techniques and extended life expectancies, the number of older adult patients with pancreatic cancer has increased substantially. Older adult patients, especially those aged 80 years or older, often have multiple comorbidities and reduced physiological function, requiring particularly careful determination of their treatment plans. While surgical resection remains the only potentially curative treatment for pancreatic cancer, there is a paucity of evidence regarding the safety and efficacy of surgical interventions among patients aged 80 years or older. In addition, most clinical trials evaluating chemotherapy treatments for cancer have excluded older adult populations over 80 years of age, which leaves a critical gap in the scientific understanding of optimal management strategies for this growing and vulnerable patient population.

The absence of robust data on surgical outcomes of pancreatic cancer among octogenarians has resulted in a lack of clear guidelines, thereby complicating the decision‐making processes for clinicians. This systematic review and meta‐analysis aim to bridge this gap by analyzing the available evidence on the efficacy and safety of surgical treatment for pancreatic cancer among patients aged 80 years or older. These findings will guide clinicians toward evidence‐based treatment decisions for older adult patients.

## 2 Methods

### 2.1 Study Design and Search Strategy

This meta‐analysis was performed in accordance with the Preferred Reporting Items for Systematic Reviews and Meta‐Analyses (PRISMA) 2020 [3]. A comprehensive literature search was performed on PubMed, the Cochrane Library database (Cochrane Central Register of Controlled Trials [CENTRAL]), and the ICHUSHI website for relevant available articles from January 1, 2010 to January 31, 2024. The search criteria were (“Pancreatic Neoplasms/surgery”[majr] AND “Aged, 80 and over”[mesh] AND (english[la] OR japanese[la]) AND 2010:2024/01[dp]), etc. The literature for undetected relevant studies and other relevant studies identified through additional manual searches were also reviewed. The search filter “Duplicate Publication” in PubMed and other databases was used to exclude duplicate papers. Ethical approval was not required because of the nature of this meta‐analysis.

### 2.2 Study Selection and Data Extraction

As a first screening, two authors (N.I. and E.M.) and one assistant (Y.S.) independently reviewed and assessed each article's title and abstract based on the following criteria.

Inclusion Criteria: (a) original materials comprising published literature discussing comparisons of the efficacy of pancreatectomy versus non‐surgical treatments among patients aged 80 years or older with pancreatic cancer, as well as comparisons of the efficacy and safety of pancreatectomy for pancreatic cancer between patients aged 80 years or older and those younger than 80 years; (b) randomized controlled trials and observational studies; (c) research sample size of 25 or more patients aged 80 years or older; (d) unlimited follow‐up time; (e) unlimited types of publication languages; (f) unlimited publication types; (g) human research; and (h) overall survival, mortality, and morbidity (postoperative complications) as research indicators.

Exclusion Criteria: (a) studies in which there was incomplete information and invalid data; studies in which the author failed to reply after being contacted; (b) non‐contrast studies; and (c) studies in which the age boundary for identifying older adult patients was not 80 years.

Two authors (N.I. and E.M.) reviewed and assessed the full text of each study that passed the first screening for inclusion in the meta‐analysis. Discrepancies between reviewers were resolved through discussions at several meetings.

The following information was extracted from the selected studies: first author, publication year, country, study design, study period, measurements of the experimental and control groups, sample size, age, gender, and outcomes. The outcomes of this meta‐analysis are overall survival, mortality, and morbidity. In cases in which the studies did not provide detailed survival data, we contacted the authors directly to request the required information. If we did not obtain a response from the authors, we extracted the survival data from the Kaplan–Meier curves presented in the studies. Disagreements between the reviewers were resolved through consultation with an additional author at the meetings.

### 2.3 Quality Assessment

Only retrospective observational studies were included in this meta‐analysis. The risk of bias in the studies was evaluated using the Newcastle–Ottawa Scale (NOS), which is structured into three parts (research population selection, comparability, and outcome evaluation) [4]. The full NOS score was nine points, and studies with scores < 6 points were excluded.

### 2.4 Data Analysis

The Mantel–Haenszel method and random‐effects models were used to assess inverse variances and 95% confidence intervals (CIs) for mean differences in categorical variables. The results were reported as odds ratios (ORs) and 95% CIs. The *I* ^2^ test was used to evaluate heterogeneity. Funnel plots were used to examine publication bias. All statistical analyses were performed using Review Manager software (version 5.4; The Nordic Cochrane Centre, The Cochrane Collaboration, 2020, Copenhagen, Denmark).

## 3 Results

### 3.1 Study Selection

**FIGURE 1:**
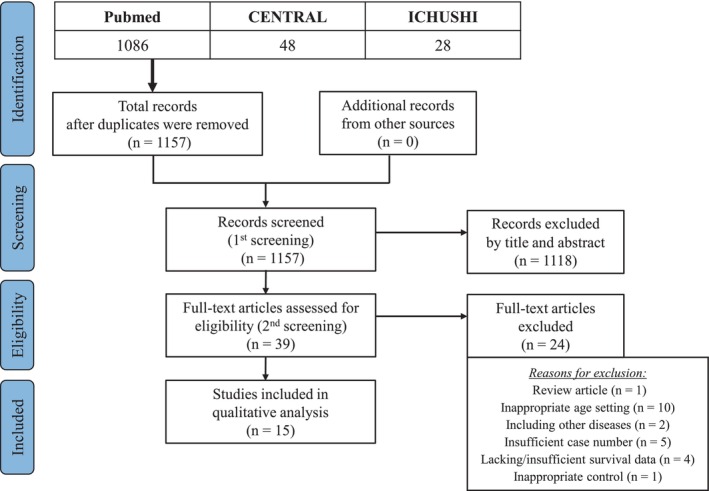
PRISMA flow chart illustrating the search strategy, excluded studies, and included studies in the quantitative analysis.

A flow diagram of this systematic review is shown in Figure 1. The database search identified 1157 studies after duplicates were removed. After the initial screening, the titles, abstracts, and full texts of 39 studies were assessed for eligibility. Finally, 15 retrospective observational studies [5, 6, 7, 8, 9, 10, 11, 12, 13, 14, 15, 16, 17, 18, 19] written in English met the inclusion criteria and were included in this review and analysis. Among them, four studies compared overall survival (OS) among patients aged 80 years or older who underwent pancreatectomy with that among those who underwent chemotherapy [9, 13, 14, 17] (Table 1). Thirteen studies evaluated OS, mortality, and/or morbidity among patients aged 80 years or older who underwent pancreatectomy compared with patients younger than 80 years [5, 6, 7, 8, 10, 11, 12, 14, 15, 16, 17, 18, 19] (Table 2). The NOS scores in these studies ranged from six to eight (Table S1).

**TABLE 1:** Characteristics and quality assessment of included studies comparing overall survival between pancreatic resection and chemotherapy among patients aged 80 years or older with pancreatic cancer.

Study	Country	Period	Case		Age		Endpoints	Quality^a^
			Pancreatectomy	Chemotherapy	Pancreatectomy	Chemotherapy		
Kinoshita 2015 [9]	Japan	2005–2012	26	20	Median (range): 82 (80–87)	Median (range): 82 (80–88)	OS	6
Satoi 2020 [13]	Japan	2007–2014	369	99	Mean (SD): 82 (2.2)	Mean (SD): 83 (2.3)	OS	8
Hue 2021 [14]	USA	2011–2016	830	1855	NA	NA	OS	6
Boutros 2023 [17]	USA	2011–2017	119	85	NA	NA	OS	6

Abbreviations: OS, overall survival; SD, standard deviation; USA, United States of America.

^a^ Indicates the scores evaluated using the Newcastle–Ottawa Scale. A detailed assessment of the enrolled studies according to the Newcastle–Ottawa Scale is provided in Table S1.

**TABLE 2:** Characteristics and quality assessment of the included studies comparing overall survival (OS), mortality, and/or morbidity between patients aged 80 years or older who underwent pancreatectomy and those younger than 80 years.

Study	Country	Period	Comparison		Age		Endpoints	Quality^a^
			Age ≥ 80	Age < 80	Age ≥ 80	Age < 80		
Khan 2010 [5]	USA	1981–2007	53	564	NA	Median (ICQ): 66 (58–72)	OS, mortality, morbidity	7
Lee 2010 [6]	USA	1992–2009	45	346	Median (ICQ): 83 (81–84)	Median (ICQ): 64 (55–71)	Mortality, morbidity	7
Melis 2012 [7]	USA	1990–2009	25	175	Mean (SD): 83.1 (2.4)	Mean (SD): 64.4 (10.3)	OS, mortality, morbidity	6
Turrini 2013 [8]	France	2004–2009	64	868	Mean (range): 83 (80–97)	NA	OS, mortality, morbidity	6
Sho 2016 [10]	Japan	2001–2012	99	1302	NA	NA	OS, mortality, morbidity	6
Sugiura 2017 [11]	Japan	2007–2014	28	199	Median (range): 82 (80–88)	NA	OS, mortality, morbidity	7
Okabayashi 2020 [16]	Japan	2005–2018	60	240	Median (range): 83 (80–91)	Median (range): 68 (18–79)	OS	7
Kondo 2020 [12]	Japan	2004–2018	56	358	NA	NA	Mortality, morbidity	8
Hue 2021 [14]	USA	2011–2016	1820	12 463	NA	NA	Mortality	6
Izumo 2021 [15]	Japan	2000–2018	31	548	Mean (range): 81 (80–87)	Mean (range): 65 (36–79)	OS, mortality, morbidity	7
Pande 2023 [19]	UK	2008–2017	110	110	Median (range): 81 (80–86)	Median (range): 69 (36–79)	OS, mortality	6
Boutros 2023 [17]	USA	2011–207	119	208	NA	NA	OS, mortality	6
Ikenaga 2023 [18]	Japan	2010–2021	25	277	NA	NA	OS	6

Abbreviations: ICQ, interquartile range; OS, overall survival; SD, standard deviation; UK, United Kingdom; USA, United States of America.

^a^ Indicates the scores evaluated using the Newcastle–Ottawa Scale. A detailed assessment of the enrolled studies according to the Newcastle–Ottawa Scale is provided in Table S1.

### 3.2 Survival Rate (Comparison Between Surgery and Chemotherapy Among Older Adult Patients)

**FIGURE 2:**
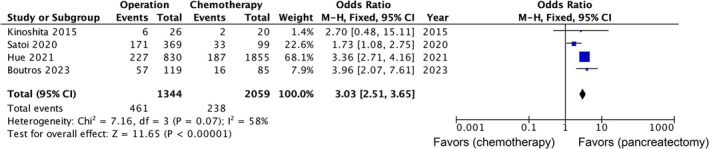
Forest plot comparing two‐year survival rates among patients aged 80 years or older who underwent pancreatectomy and chemotherapy for pancreatic cancer. The solid squares denote the odds ratios. The horizontal lines represent the 95% CIs, and the diamond denotes the pooled effect size. M‐H, Mantel–Haenszel test.

The four studies [9, 13, 14, 17] shown in Table 1 included 1344 patients aged 80 years or older who underwent pancreatectomy and 2059 patients aged 80 years or older who received chemotherapy instead of surgical treatment. Data on two‐year survival from one study [9] were directly obtained from the original author. The rates of two‐year survival after pancreatectomy and chemotherapy were 34.3% and 11.6%, respectively. Patients aged 80 years or older who underwent pancreatectomy had a higher two‐year survival rate than those who were treated with chemotherapy (OR: 3.03, 95% CI: 2.51–3.65, *p* < 0.00001; Figure 2). The heterogeneity among the studies was moderate (*I* ^2^ = 58%, *p* = 0.07).

### 3.3 Survival Rate (Comparison Between Older Adult and Younger Patients)

**FIGURE 3:**
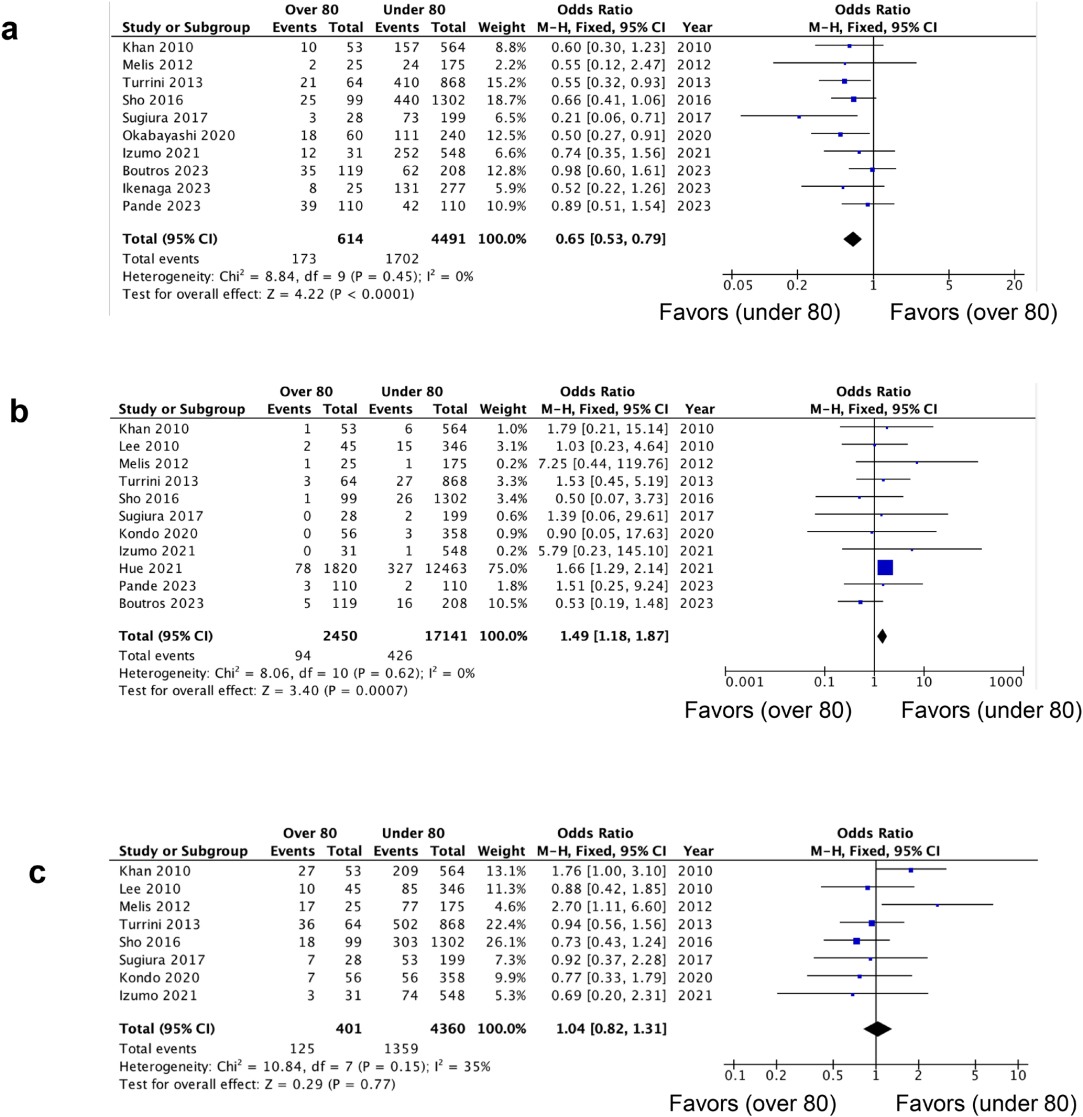
Forest plots of (a) three‐year survival, (b) mortality, and (c) morbidity comparing patients aged 80 years or older with patients younger than 80 years. The solid squares denote the odds ratios. The horizontal lines represent the 95% CIs, and the diamond denotes the pooled effect size. M‐H, Mantel–Haenszel test.

Ten studies [5, 7, 8, 10, 11, 15, 16, 17, 18, 19] among those presented in Table 2 reported OS in 614 patients aged 80 years or older who underwent pancreatectomy and 4491 patients younger than 80 years who underwent pancreatectomy for pancreatic cancer. Three‐year survival data were directly obtained from the original author in three studies [11, 15, 19] and extracted from the Kaplan–Meier curves in three studies [5, 7, 10]. The three‐year survival rates among older adults and younger patients were 28.2% and 37.9%, respectively. Patients aged 80 years or older had a lower three‐year survival rate than those younger than 80 years (OR: 0.65, 95% CI: 0.53–0.79, *p* < 0.0001; Figure 3A). No heterogeneity was observed in the reported data (*I* ^2^ = 0%; *p* = 0.45).

### 3.4 Mortality (Comparison Between Older Adult and Younger Patients)

Eleven studies [5, 6, 7, 8, 10, 11, 12, 14, 15, 17, 19] reported mortality. Mortality among patients aged 80 years or older was significantly higher than that among younger patients (OR: 1.49, 95% CI: 1.18–1.87, *p* = 0.0007; Figure 3B). Heterogeneity between the studies was not observed (*I* ^2^ = 0%, *p* = 0.62).

### 3.5 Morbidity (Comparison Between Older Adult and Younger Patients)

Eight studies [5, 6, 7, 8, 10, 11, 12, 15] reported morbidity. Morbidity corresponding to complications of Clavien–Dindo classification grade III or higher [20] was reported in five studies [6, 10, 11, 12, 15]. Other studies [5, 7, 8] also reported morbidity. There is no significant difference in morbidity between older adult patients and younger patients (OR: 1.04, 95% CI: 0.82–1.31, *p* = 0.77; Figure 3C). The heterogeneity among the studies was low (*I* ^2^ = 35%, *p* = 0.15).

### 3.6 Publication Bias

**FIGURE 4:**
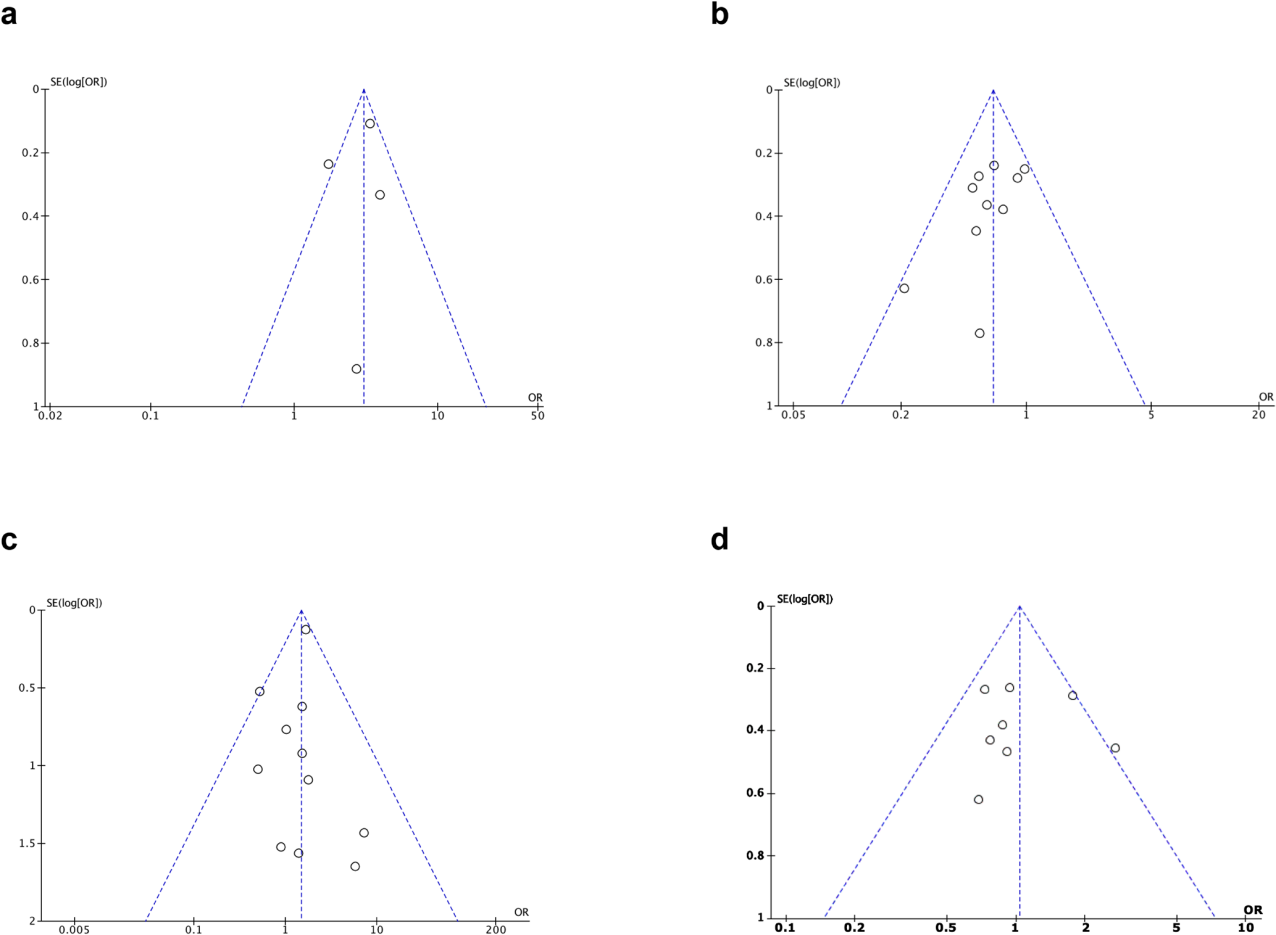
(a) Funnel plot of comparison in two‐year survival of patients aged 80 years or older who underwent pancreatectomy and chemotherapy for pancreatic cancer. Funnel plots of comparison in (b) three‐year survival, (c) mortality, and (d) morbidity comparing patients aged 80 years or older with patients younger than 80 years.

Funnel plots of the ORs in the analyzed studies were essentially symmetrical for all endpoints (Figure 4). The possibility of publication bias was low.

## 4 Discussion

With an aging global population, there has been increasing interest in the safety and effectiveness of surgical treatment for pancreatic cancer among older adult patients. However, no systematic review or meta‐analysis had previously been conducted to evaluate these outcomes among patients aged 80 years or older. Our meta‐analysis revealed that pancreatectomy offers better prognosis than chemotherapy alone among older adult patients with pancreatic cancer. However, we also found that patients in this age group experienced higher postoperative mortality and slightly lower three‐year survival rates than younger patients, which likely reflects the impact of age‐related health issues and other conditions that are common among older adults. This meta‐analysis is the first to provide comprehensive results from the available evidence regarding the surgical treatment for pancreatic cancer in this specific patient group.

Surgical resection is the only curative treatment for pancreatic cancer without clear distant metastasis [21]. Previous randomized controlled trials and retrospective studies have shown that surgical treatment results in a significantly higher survival rate among patients with pancreatic cancer than nonsurgical treatment [22, 23]. However, pancreatic resection, especially pancreaticoduodenectomy, is one of the most complex and invasive surgeries with high morbidity and mortality rates [24, 25]. Therefore, whether pancreatic resection is an effective treatment for pancreatic cancer in older adult patients, who are thought to have reduced physical strength, has been a hot topic in recent years. An analysis of a small number of cases reported in 2015 demonstrated that surgical resection did not improve prognosis compared with chemotherapy among patients with pancreatic cancer aged 80 years or older (MST: 12.5 vs. 11.7 months, *p* = 0.263) [9]. This meta‐analysis including four studies has revealed that patients aged 80 years or older who underwent pancreatic surgery had a significantly higher two‐year survival rate than those who received chemotherapy (OR: 3.03, 95% CI: 2.51–3.65). This finding suggests that surgical intervention may provide a substantial benefit even among older adult patients and challenges the notion that advanced age should be a reason to avoid surgery. Nonetheless, it is important to carefully evaluate each patient's health status and risks before making decisions regarding surgical treatment because older patients may find it more difficult to recover from the procedure.

To assess the usefulness and safety of surgical treatment among older adult patients, perioperative outcomes are often compared with those involving younger patients. Although comparing older adult and younger patients is not always meaningful when deciding treatment for older adults, it can serve as a useful reference for estimating the safety of surgery. This meta‐analysis revealed that postoperative mortality among older adult patients was higher than that among younger patients (OR: 1.49, 95% CI: 1.18–1.87). This highlights the greater difficulties faced by patients aged 80 years or older during and after surgery. In terms of postoperative complications, there was no significant difference in the morbidity rate between older adult and younger patients (OR, 1.04; 95% CI: 0.82–1.31). This suggests that, compared to younger patients, older adults may have a reduced ability to recover from complications, leading to a higher likelihood of fatal outcomes. This can be attributed to diminished physiological reserves and the presence of comorbidities among patients aged 80 years or older, which impair their capacity to overcome postoperative complications. The estimated mortality rate of 3.8% among patients aged 80 years or older in this analysis is not especially high compared to the mortality rate of pancreatectomy from previous reports, and it is not significant enough to justify avoiding surgical resection as a treatment option. However, older adult patients require careful preoperative evaluation and postoperative care.

Previous studies comparing postoperative outcomes between older adult and younger patients with pancreatic cancer using 80 years as the threshold have produced mixed results regarding prognosis. Some studies have reported poorer outcomes among patients aged 80 years or older, whereas others have found no significant differences. In the current meta‐analysis, which included 10 studies, six reported no significant difference in survival between the two age groups [5, 7, 8, 15, 17, 19], whereas four reported worse outcomes among patients aged 80 years or older [10, 11, 16, 18]. The pooled OR for three‐year survival in these studies demonstrated that older adult patients had a lower survival rate than younger patients. This finding suggests that while pancreatic resection remains a valid treatment option for older adult patients, its survival advantage may be reduced compared with that among younger patients. The decision for surgery among patients aged 80 years or older should be weighed carefully, considering their individual risk factors and potential benefits. In the four studies that reported worse prognoses among older adult patients, a common factor identified in this population was the lower rate of administration and/or completion of adjuvant chemotherapy in comparison with younger patients. In a multivariate analysis of prognostic factors among older adult patients, Sho et al. [10] found that the completion of adjuvant chemotherapy was the only significant factor influencing survival. Ikenaga et al. [18] revealed that patients aged 80 years or older who received perioperative chemotherapy had improved outcomes compared with those who did not, with OS approaching that of younger patients. These findings support the active consideration of adjuvant chemotherapy even among patients aged 80 years or older. However, Okabayashi et al. [16] reported that only 60% of patients aged 80 years or older who underwent surgery died from cancer recurrence, with a higher proportion of pancreatic cancer‐unrelated deaths compared to younger patients. The annual death rate for individuals aged 80 years or older is estimated to be over 4.9% in Japan and increases with aging. Older adult patients with a poor performance status who cannot undergo chemotherapy may need to be considered for exclusion from surgical indications.

This study had several limitations. First, all the included studies were retrospective observational studies, and this may have introduced selection and information biases. Patients who underwent surgery were likely healthier than those who received chemotherapy, which limited the direct comparison between the two treatment groups. However, considering the nature of the topic, randomized controlled trials are difficult to conduct. Future prospective registry studies using propensity score matching may provide more reliable and high‐quality results. Second, data from the included studies were collected over a 40‐year period (1981–2021) from a heterogeneous patient cohort. Perioperative management of patients and therapeutic strategies for pancreatic cancer have advanced dramatically during this period. The findings obtained in this study may not apply to current practices in the treatment of pancreatic cancer. Third, there was a limited number of studies available comparing the outcomes of surgery and chemotherapy in older adult patients with pancreatic cancer. This scarcity of relevant data constitutes another important limitation of the present review, underscoring the need for more focused research in this area. Regarding the sample size criterion, we set a threshold of 25 older adult patients or more to ensure the statistical power of the studies included. Smaller sample sizes may result in unreliable estimates, which could affect the validity and generalizability of the results. This threshold was chosen based on common practices in systematic reviews and meta‐analyses to ensure the robustness of our findings. Finally, the included literature originates from several countries. Variations in healthcare systems, surgical techniques, and experience across different regions may have influenced the results. Although all the literature has been reported in developed countries, there is still a disparity in the safety of pancreatectomy between Japan and Western countries [26, 27, 28]. These findings must be interpreted in the context of each country's healthcare system for older adults.

In conclusion, surgical treatment improves survival rates compared to chemotherapy alone even among patients aged 80 years or older with pancreatic cancer. Compared with patients under the age of 80 years, the older age group experiences higher perioperative mortality and shorter postoperative survival rates. These objective findings should be clearly presented to patients to allow informed and shared decision‐making when determining the most appropriate treatment strategy.

## Author Contributions

Conceptualization and original draft: Naoki Ikenaga and Takao Ohtsuka. Methodology, data curation, and formal analysis: Naoki Ikenaga, Eiji Mitate, and Koji Tamura. Investigation and data resources: Naoki Ikenaga, Eiji Mitate, and Masayuki Sho. Project administration: Takao Ohtsuka and Masafumi Nakamura. Supervision: Masafumi Nakamura. Critical review and final approval of manuscript: All authors.

## Conflicts of Interest

The authors declare no conflicts of interest.

## Supporting information


Table S1.

## Data Availability

The data that support the findings of this study are available from the corresponding author upon reasonable request.
