# Supplementary material for: Efficacy and Safety of Surgical Treatment Among Older Adult Patients (80 Years) With Pancreatic Cancer: A Systematic Review and Meta‐Analysis
Source: J Hepatobiliary Pancreat Sci. 2025 May 14;32(7):497–505. doi: 10.1002/jhbp.12151 (PMC12276457; doi:10.1002/jhbp.12151)
Supplement: Supplementary file 1 — Table S1. [file JHBP-32-497-s001.docx]

**Supplementary Table 1.** Newcastle-Ottawa Scale assessment of the enrolled studies.

| Study | Selection | | | | Comparability | | Outcome | | | Total |
| --- | --- | --- | --- | --- | --- | --- | --- | --- | --- | --- |
|  | 1 | 2 | 3 | 4 | 5 | 6 | 7 | 8 | 9 |  |
| Khan 2010 | ☆ | ☆ | ☆ | ☆ |  |  | ☆ | ☆ | ☆ | 7 |
| Lee 2010 | ☆ | ☆ | ☆ | ☆ |  |  | ☆ | ☆ | ☆ | 7 |
| Melis 2012 | ☆ | ☆ | ☆ | ☆ |  |  | ☆ | ☆ |  | 6 |
| Turrini 2013 | ☆ | ☆ | ☆ | ☆ |  |  | ☆ | ☆ |  | 6 |
| Kinoshita 2015 | ☆ | ☆ | ☆ | ☆ |  |  | ☆ | ☆ |  | 6 |
| Sho 2016 | ☆ | ☆ | ☆ | ☆ |  |  | ☆ | ☆ |  | 6 |
| Sugiura 2017 | ☆ | ☆ | ☆ | ☆ |  |  | ☆ | ☆ | ☆ | 7 |
| Okabayashi 2020 | ☆ | ☆ | ☆ | ☆ |  |  | ☆ | ☆ | ☆ | 7 |
| Satoi 2020 | ☆ | ☆ | ☆ | ☆ | ☆ | ☆ | ☆ | ☆ |  | 8 |
| Kondo 2020 | ☆ | ☆ | ☆ | ☆ |  | ☆ | ☆ | ☆ | ☆ | 8 |
| Hue 2021 | ☆ | ☆ | ☆ | ☆ |  |  | ☆ | ☆ |  | 6 |
| Izumo 2021 | ☆ | ☆ | ☆ | ☆ |  |  | ☆ | ☆ | ☆ | 7 |
| Pande 2023 | ☆ | ☆ | ☆ | ☆ |  |  | ☆ | ☆ |  | 6 |
| Boutros 2023 | ☆ | ☆ | ☆ | ☆ |  |  | ☆ | ☆ |  | 6 |
| Ikenaga 2023 | ☆ | ☆ | ☆ | ☆ |  |  | ☆ | ☆ |  | 6 |

Note: 1. Representativeness of the exposed cohort; 2. Selection of the non-exposed cohort; 3. Ascertainment of exposure; 4. Outcome of interest was not reported at the beginning of the study; 5. Study controls for age, gender, and marital status; 6. Study controls for any additional factors; 7. Assessment of outcomes; 8. Follow-up long enough for outcomes to occur; and 9. Adequacy of follow-up.
